# Parliament and Publications

**Published:** 1911-03-18

**Authors:** 


### PARLIAMENT AND PUBLICATIONS.

### THE PLAGUE IN EAST ANGLIA.

Mr. John Burns stated in the House of Commons that since the four suspected cases of human plague which occurred in East Anglia in September last no further cases have been observed, but a certain number of rats and rabbits have been found to be infected with plague.

### MEDICAL PRACTITIONERS AND THE PETROL DUTY.

Rebate on the motor spirit duty is refused unless the Commissioners of Customs and Excise are satisfied that duty has, in fact, been paid to the Crown in respect of the quantities used by claimants of allowance of duty. This statement was made by Mr. Hobhouse in reply to Mr. Ginnell, who asked a question on behalf of medical practitioners.

### WHITE BREAD (PHYSICAL DEGENERACY).

In reply to a question by Mr. Charles Bathurst, Mr. Trevelyan stated that the medical officers of the Board of Education had expressed no opinion as to the relation between the consumption of bread of any particular character and the decay of the teeth. The best hope of arresting tendencies to physical degeneration appeared to the Board to lie in the development of the school medical service and of school hygiene in its various aspects.

### GENERALISED VACCINIA.

With reference to the care of a child who died from broncho-pneumonia supervening on generalised vaccinia after having been vaccinated with lymph supplied by the Local Government Board, Mr. John Burns stated, in reply to a question, that the lymph in question belonged to a series of which 1,629 tubes were despatched, and no complaints had been received in regard to other cases for which the tubes were used. Although during the two years, 1909 and 1910, over one million vaccinations were performed, only two cases of generalised vaccination are known to have occurred.

### NURSES' REGISTRATION.

The Bill introduced in the House of Commons by Mr. Munro Ferguson has now been printed. It provides for the establishment of a council called "the General Council for the Registration of Nurses in the United Kingdom," consisting of twenty-one persons. Three members of the Council (of whom one at least shall be a woman) are to be appointed by the Privy Council; the Local Government Boards for England, Scotland, and Ireland are each to appoint one registered medical practitioner; the British Medical Association is to appoint three registered medical practitioners; one practitioner is to be appointed by the Medico-Psychological Association, and another by the medical superintendents of fever hospitals. Eight registered women nurses are to be elected as the direct representatives of registered women nurses. Male nurses are to have one representative, and mental nurses another. On the first constitution of the Council, however, in the place of the direct representation of registered nurses, fourteen persons are to be appointed to hold office until the task of forming a register is sufficiently advanced to admit of an election of direct representatives. Provision is made for the registration without examination of trained nurses during a period of three years, but at the expiration of that term persons claiming to be registered must have had not less than three years' training under a definite curriculum prescribed by the Council in the wards of a hospital approved of by the Council or in an institution recommended by the Local Government Board, or must have been trained under regulations authorised by the Lords Commissioners of the Admiralty or the Army Council.

